# Neonatal jaundice detection in low-resource Mexican settings: possibilities and barriers for innovation with mobile health

**DOI:** 10.1186/s12913-024-11141-6

**Published:** 2024-05-28

**Authors:** Gabriela Jiménez-Díaz, Anders Aune, Jesús Elizarrarás-Rivas, Lobke M. Gierman, Martina Keitsch, Anna Marcuzzi, Jennifer J. Infanti

**Affiliations:** 1 Department of Public Health and Nursing, Faculty of Medicine and Health Sciences, NTNU-Norwegian University of Science and Technology, Trondheim, Norway; 2 Picterus AS, Trondheim, Norway; 3 Health Research Coordination, Mexican Institute of Social Security, IMSS, Oaxaca, Mexico; 4 Faculty of Medicine and Surgery, Universidad Autónoma Benito Juárez de Oaxaca, Oaxaca City, México; 5 Department of Design, Faculty of Architecture and Design, Norwegian University of Science and Technology, Trondheim, Norway; 6 Department of Physical Medicine and Rehabilitation, St. Olav’s University Hospital, Trondheim, Norway; 7 Department of Pediatrics, St Olav’s University Hospital, Trondheim, Norway

**Keywords:** Neonatal jaundice, Hyperbilirubinemia, mHealth, Screening, Detection, Implementation, Low- and middle-income countries, Healthcare systems

## Abstract

**Background:**

Neonatal jaundice is a common condition that can lead to brain damage and disabilities when severe cases go undetected. Low- and middle-income countries often lack accurate methods for detecting neonatal jaundice and rely on visual assessment, resulting in a higher incidence of adverse consequences. Picterus Jaundice Pro (Picterus JP), an easy-to-use and affordable smartphone-based screening device for the condition, has demonstrated higher accuracy than visual assessment in Norwegian, Philippine and Mexican newborns. This study aimed to identify the barriers and facilitators to implementing Picterus JP in public health services in low-income settings in Mexico by exploring the current process of neonatal jaundice detection and stakeholders’ perspectives in that context.

**Methods:**

Qualitative data collection techniques, including one focus group, 15 semi-structured interviews and four observations, were employed in urban and rural health facilities in Oaxaca, Mexico. The participants included medical doctors, nurses and health administrators. The data were analysed by thematic analysis guided by the Consolidated Framework for Implementation Research.

**Results:**

The analysis yielded four main themes: (I) the current state of neonatal care and NNJ detection, (II) the needs and desires for enhancing NNJ detection, (III) the barriers and facilitators to implementing Picterus JP in the health system and (IV) HCWs’ expectations of Picterus JP. The findings identify deficiencies in the current neonatal jaundice detection process and the participants’ desire for a more accurate method. Picterus JP was perceived as easy to use, useful and compatible with the work routine, but barriers to adoption were identified, including internet deficiencies and costs.

**Conclusions:**

The introduction of Picterus JP as a supporting tool to screen for neonatal jaundice is promising but contextual barriers in the setting must be addressed for successful implementation. There is also an opportunity to optimise visual assessment to improve detection of neonatal jaundice.

**Supplementary Information:**

The online version contains supplementary material available at 10.1186/s12913-024-11141-6.

## Background

Neonatal jaundice (NNJ) affects 60–80% of newborns within the first 48 to 72 h of life [1]. Due to increased production and decreased elimination of bilirubin in newborns, it accumulates in the blood, causing hyperbilirubinemia [2]. While most cases resolve without consequences, severe NNJ affects approximately 1.1 million infants annually, necessitating close monitoring and treatment [3]. If undetected, severe hyperbilirubinemia can lead to brain damage and conditions such as acute bilirubin encephalopathy, impaired neurologic development, long-term disabilities (including audiologic, language processing, cognitive and visual-motor disturbances) and, in the worst cases, death [4–6].

Currently, reliable detection of NNJ depends on invasive or expensive methods, such as transcutaneous bilirubinometer (TcB) devices and total serum bilirubin (TSB) blood tests, which are limited in most low- and middle-income countries (LMICs) as well as in non-hospital settings [7–9]. As a result, detection relies on visual assessment (VA), an unreliable and inaccurate screening method [10, 11]. This approach deviates from the recommendations found in most clinical practice guidelines (CPGs), which advise measuring bilirubin either with TcB or TSB in all newborns before hospital discharge after birth and in all newborns older than 24 h with suspected jaundice [12–14]. The reliance on VA leads to a higher prevalence of severe consequences of NNJ in LMICs than in high-income countries (HICs) [1, 15, 16]. This results in preventable health inequities with significant social and economic costs.

**Fig. 1:**
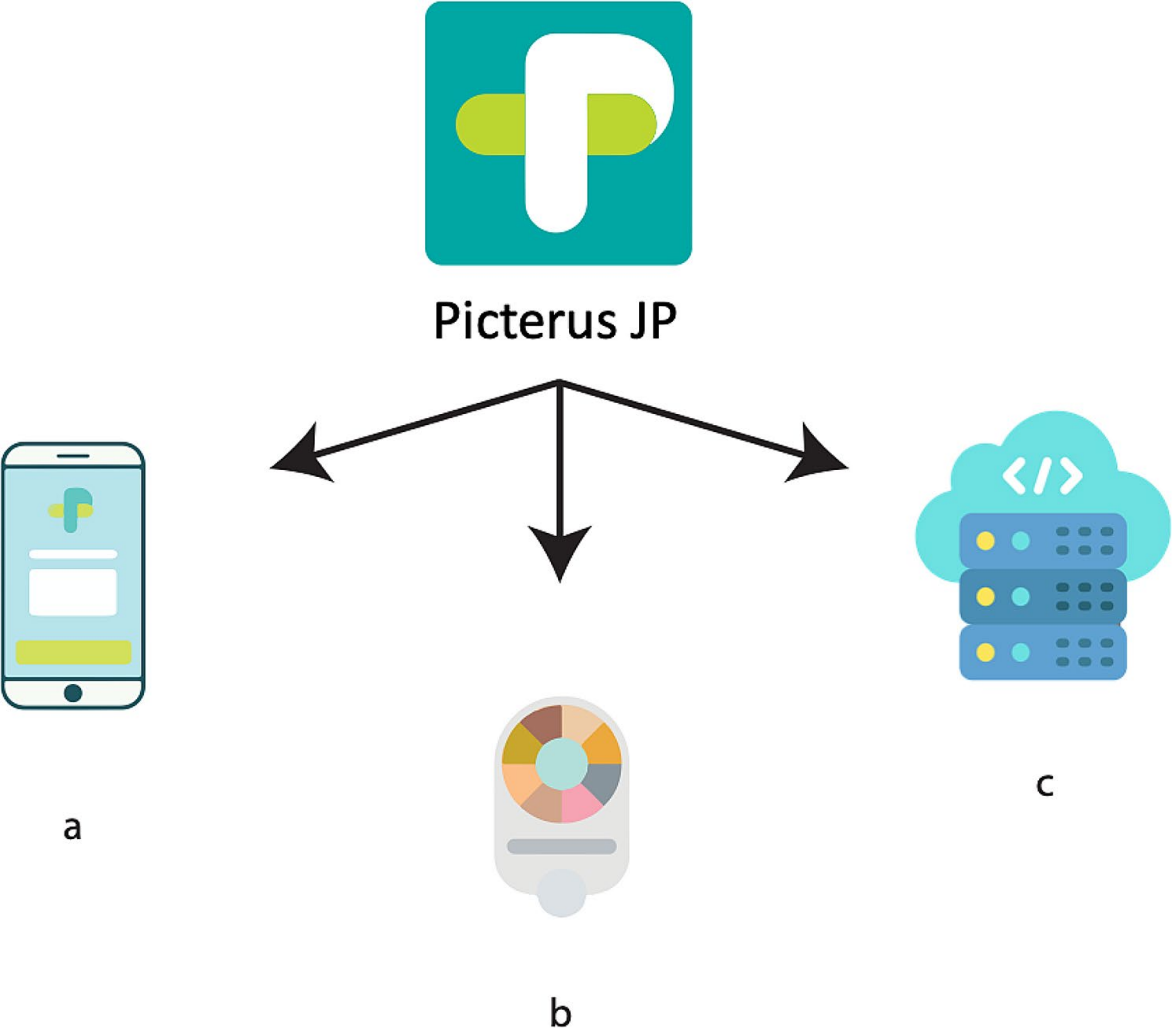
Picterus JP components: **a**. smartphone app; **b**. colour calibration card; **c**. server

In response to these challenges, a Norwegian multidisciplinary research group developed an accessible, affordable mobile health (mHealth) solution called Picterus Jaundice Pro (Picterus JP) to screen for NNJ. Picterus JP consists of a smartphone app to capture and upload images of the newborn’s skin, a calibration card for accurate colour calibration in the images and a server for image analysis and storage (Fig. 1) [17]. The app has undergone validation in Norwegian hospitals [17, 18] and in pilot studies in Mexico and the Philippines [19, 20], demonstrating significant positive correlation between Picterus JP*’s* bilirubin values and TSB levels as well as higher accuracy over VA.

Digital health technologies such as Picterus JP have immense potential to enhance healthcare in resource-constrained areas, but such innovations face implementation challenges that can hinder their adoption, leading to slow, unreliable and unsustainable outcomes, particularly in LMICs, such as Mexico [21]. Implementation factors, including the setting, stakeholder considerations and the innovation characteristics, play a crucial role in achieving successful outcomes [22].

Statistics on NNJ in Mexico are limited, with scarce hospital-based studies, and this lack of data prevents precise national estimates. A study conducted in the neonatal intensive care unit (NICU) of one Mexican hospital reported a NNJ prevalence of 17% in 2008 [23]. Another study documented 127 newborns with NNJ admitted to a general hospital over a five-year period [24]. However, a study that followed 156 infants with sensorineural hearing loss who had experienced a NICU stay with multiple diagnoses including NNJ, highlighted severe hyperbilirubinemia as the primary risk factor for hearing loss, indirectly indicating a significant yet unreported burden of the disease [25]. While Mexico has CPGs for the diagnosis and treatment of NNJ, which were updated in 2019 [13], there are no reports regarding their utilisation in actual clinical practice.

The present study aimed to identify barriers and facilitators to implementing Picterus JP in public health services in low-income settings in Mexico by exploring current NNJ practices in the context along with stakeholder requirements for enhancement and their perceptions of using the device. The findings provide insights into the feasibility of implementing Picterus JP as an innovative future intervention for NNJ detection in the setting.

## Methods

### Study design

A qualitative approach was employed to effectively address the study aim and provide a comprehensive understanding of neonatal care services, contextual factors and the feasibility of implementing Picterus JP to screen for NNJ in the context. Observations, semi-structured interviews and focus group were chosen to obtain in-depth insights and diverse perspectives [26].

### Study setting

Mexico exhibits significant urban-rural inequalities, with 43.9% of the population living below the income poverty line and 8.5% in extreme poverty as of 2020 [27]. Income disparities are pronounced between the wealthy northern states and impoverished southern ones [28]. The Mexican health system comprises public and private providers overseen by the Ministry of Health and includes employment-based social insurance schemes, public assistance services with financial protection for the uninsured and private-sector schemes. However, widespread workforce shortages and resource inconsistencies limit access to services and innovative technologies, particularly in rural areas [29].

The study was conducted in Oaxaca, one of Mexico’s poorer states, at both urban and rural healthcare facilities affiliated with Instituto Mexicano del Seguro Social (IMSS), the largest social welfare institution in Latin America [28, 30, 31]. The selection of diverse sites allowed for the recruitment of participants serving populations with varied backgrounds and socioeconomic situations.

### Participant recruitment

Purposive sampling was used to recruit health administrators and healthcare workers (HCWs) with diverse educational and working backgrounds, specifically doctors and nurses involved in providing newborn care or connected to neonatal care services. Prior to formal data collection, the project’s details, data collection methodology and inclusion criteria were explained to staff members during introductory meetings at each study site. The voluntary nature of participation was emphasised and interested individuals provided their contact information. The first author directly contacted the health administrators.

### Data collection

#### Observations

The first author made non-participant observations, i.e., without active participation in the observed activities and interactions, while HCWs performed their daily activities related to neonatal care. This approach enabled direct observation of the neonatal care services in action, offering valuable contextual information.

### Semi-structured interviews

The semi-structured interviews facilitated open, flexible conversations with stakeholders, including HCWs and administrators, to explore their experiences, needs and perceptions regarding the implementation of Picterus JP. They were conducted in person except for one by video call. The interviews were conducted and audio-recorded in Spanish using a semi-structured question guide showed in an additional file (see Additional file 1) at the participants’ work facilities during their shifts. Basic demographic information was collected, such as age, gender, education level and years of healthcare experience, but names were not requested.

The interviews consisted of two parts. The first focused on the participants’ current workload, the process of NNJ detection and their identified needs for improvement. Subsequently, they were asked to test Picterus JP on a dummy, following the instructions provided with the device. The second part of the interview took place immediately after the testing. In it, various topics were discussed, including the participants’ general opinions on Picterus JP, its perceived usefulness, the factors influencing its adoption and implementation in their work routines and the healthcare system and any suggested features or changes that could enhance its optimisation. The interviews, including the testing of Picterus JP, had an average duration of 45 min.

Additionally, one focus group discussion was conducted in one of the sites using the same semi-structured question guide and general interview procedure to facilitate interactions among the participants, enabling the exploration of shared experiences and differing viewpoints. The discussion was audio-recorded and lasted 80 min.

### Data analysis and conceptual framework

To process the study data, the observation notes were translated from Spanish to English and the audio-recorded interviews and focus group discussion were transcribed verbatim in Spanish and later translated into English by the first author.

The Consolidated Framework for Implementation Research (CFIR) [32] was adopted as a theoretical framework to guide the thematic analysis of the qualitative data collected from various participants. The CFIR, a well-established framework used extensively in implementation science, provides a comprehensive set of constructs that facilitate the assessment and understanding of factors that significantly influence the successful implementation of innovations in diverse settings. With its interactive domains and multiple constructs, the CFIR encompasses several aspects of implementation outcomes, including the characteristics of the innovation, the external (outer) and internal (inner) settings and the individuals involved in the implementation process [22, 32]. Thematic analysis, known for its flexibility and practicality, was employed to code the data and identify patterns across various themes. By adopting a theory-driven approach, we conducted a detailed analysis of specific aspects of the data [33].

Initially, the first author reviewed the entire dataset, identifying keywords, concepts and perspectives relevant to the study’s objectives. These elements were selected based on their unique qualities or their frequent mention by multiple participants.

Illustrative quotes in the data were also identified at this stage. Subsequently, initial codes were generated and then categorised based on the most appropriate CFIR construct. All relevant factors in the study data that could potentially impact the implementation of Picterus JP were identified and mapped to their corresponding CFIR domain as shown in detail in an additional file (see Additional file 2). In this manner, we systematically used the CFIR to code, categorise and analyse the contextual factors operating at different levels within the setting, which are crucial to understanding the key determinants of implementation.

**Fig. 2:**
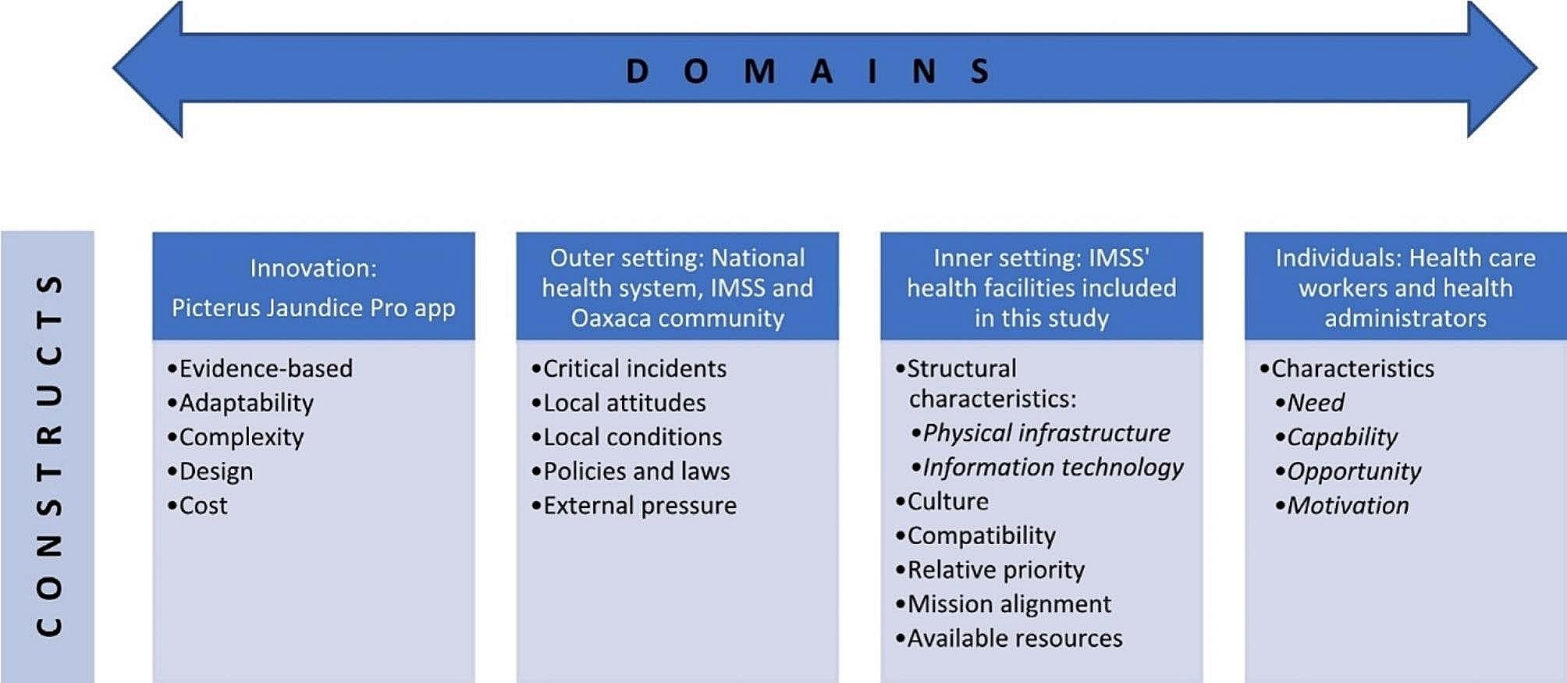
CFIR domains and constructs identified in the study (based on Damschroder, L.J., et al. The updated Consolidated Framework for Implementation Research based on user feedback. Implementation Sci 17, 75 (2022)) [29]

Figure 2 provides an overview of the CFIR domains, their specific descriptions as related to this study and the identified constructs derived from our data.

The analysis was then reviewed by two other members of the research team, and any disagreements were resolved through joint discussion sessions.

## Results

We conducted four non-participant observations, 15 semi-structured interviews and one focus group with four participants in January and February 2023. The participants included four medical doctors, 13 nurses and two health administrators with an average work experience of 13.8 years (range: 1–27 years). Table 1 provides an overview of the participant sample and characteristics for each data collection technique.

**Table 1:** Overview of participant characteristics for each data collection technique

Data collection technique	Site and number of participants	Gender	Position	Average age (range)
Non-participant observations *N* = 4	Urban clinic *n* = 2	F (2)	Family medicine nurses	Missing
	Rural hospital *n* = 2	F (2)	Preventive medicine nurses	46.5 (46–47)
Semi-structured interviews *N* = 15	Urban clinic *n* = 6	F (4) M (2)	Family medicine nurses (4) Family medicine doctors (2)	44.5 (32–56)
	Rural hospital *n* = 7	F (7)	Preventive medicine nurses (2) Outpatient clinic nurses (2) SNCU nurse (1) Neonatologist (1) Health administrator (1)	
	Urban hospital *n* = 1	M (1)	Pediatrician	
	National Medical Center *n* = 1	M (1)	Health administrator	
Focus group *N* = 1	Urban clinic *n* = 4	F (2) M (2)	Community nurses	31.5 (26–32)

F = female; M = male; SNCU = Special neonatal care unit

This section describes the four main themes identified in the collected data: (I) the current state of neonatal care and NNJ detection, (II) the needs and desires for enhancing NNJ detection, (III) the barriers and facilitators to implementing Picterus JP in the health system and (IV) HCWs’ expectations of Picterus JP.

### The current state of neonatal care and NNJ detection

The observations and interviews yielded insights into the contrasting perspectives and experiences of HCWs in the urban and rural study settings, particularly nurses, concerning their workload and the quality of care provided to newborns. Nurses and, occasionally, family medicine doctors play the primary role in neonatal care and NNJ detection at the outpatient health facilities. Nurses are responsible for identifying abnormal findings in newborns and referring them to doctors within either the outpatient clinic or the emergency room (ER). In the urban clinic, specialised nurses in family medicine work eight-hour shifts, providing care to up to four newborns per week in 16 consulting rooms. Each mother/newborn pair receives approximately 30–45 min of dedicated care and assessment. In contrast, the rural hospital’s preventive medicine unit is staffed by two permanent nurses working eight-hour shifts who, among other tasks, provide care to 8–12 newborns per day. Due to the higher number of patients, the nurses spend around 10–15 min with each mother/newborn pair. These findings are summarised and compared in Table 2.

**Table 2:** Neonatal care comparison between urban and rural facilities

Characteristics	Urban	Rural
Consulting rooms to detect NNJ	16	1
Number of nurses	16	2
Attended newborns	3–4/week	8–12/day
Time spent with each mother/baby	30–45 min	10–15 min

Interviewed nurses described their involvement in delivering various neonatal care services, including immunisation, recommendations on breastfeeding technique, neonatal metabolic screening and assessment of jaundice. Moreover, they offer guidance to mothers, advising them to expose their babies to sunlight if they observe signs of jaundice or to seek hospital care when necessary. It was observed that there was no specific standardised method used to conduct VA of NNJ and that both the urban and rural settings lacked systematic record-keeping for newborns requiring further care, which was confirmed by the interviewed participants. A rural nurse indicated that visual cues guided her decision-making in the assessment process: *‘I look at the face, chest, hands. If I see the baby is very yellow on the face, then I uncover the whole body’.*

While nurses in the urban clinic described having a manageable workload with sufficient time to carry out their activities, nurses in the rural hospital expressed a desire for more time to provide enhanced care. However, they also indicated their adaptability and dedication in managing their workload. As one nurse said, *‘We have a heavy workload, but we are already adapted to it. … although we have a lot of children [to attend to], we dedicate the necessary time to each of them to detect any anomaly in time’.*

### Needs and desires to enhance NNJ detection

The interviews made clear that NNJ detection is a routine task for HCWs, who all mentioned the importance of early identification to prevent severe consequences. While all the doctors believed that they possessed an adequate level of knowledge about NNJ, most nurses expressed concerns about the lack of updated information and training on NNJ from their academic and working institutions. This was clearly exemplified by an urban nurse who expressed: *‘We are not updated; I feel that we are behind in some respects, for example, there are mothers that tell us that paediatricians tell them that sunbathing should not be done because of the ultraviolet rays. And I can’t refute that because I don’t have the current knowledge’.*

When asked about the national guidelines for the diagnosis and treatment of NNJ, which are publicly accessible, very few HCWs were aware of their existence or had read them.

Most nurses acknowledged their lack of complete confidence in VA, and even the most experienced staff members expressed the need for a more objective method to detect NNJ. One urban nurse stated, *‘I think it would be very useful having an additional tool to detect NNJ, because we just use a colour scale, and it is very personal. Maybe I do not see the same [colour] as another person. Technology would help us’*.

Rural nurses also highlighted the issue of long waiting times for newborns referred to the ER for further care. Some mothers, unwilling to wait, leave the hospital before their newborns are evaluated.

### Barriers and facilitators to implementing picterus JP in the health system

The data generated several insights into factors that, based on the CFIR, could either drive or hinder implementation. They are presented according to the four domains of (A) innovation, (B) outer setting, (C) inner setting and (D) individuals (Table 3) and described below.

**Table 3:** Facilitators and barriers identified by stakeholders for implementing Picterus JP, based on the Consolidated Framework for Implementation Research (CFIR)

CFIR domain	Facilitators	Barriers
a) Innovation: Picterus JP	Easy to use Useful: improves detection, decreases blood pricking, saves resources	Doubts or uncertainties about performance in real-life scenarios and in all types of skin colours Requires internet connection Potential high cost
b) Outer setting: National health system, IMSS, and Oaxaca community	Aligns with mission Adequate digital literacy among healthcare workers Community acceptance through awareness-raising and dissemination would facilitate acceptance	Long process to get regulatory approval Added value needs to be identified by authorities Unstable mobile data network in Oaxaca
c) Inner setting: IMSS health facilities in the study	Adequate physical infrastructure Requires minimal equipment Compatible with work routine	Insufficient internal internet network
d) Individuals: HCWs, health administrators	Expressed need and desire to improve NNJ detection Willingness and motivation to use new technology	Resistance to change

### A. Picterus JP as an innovative tool

Overall, the participants found Picterus JP easy to use, with clear, understandable and practical instructions and a user-friendly design. They particularly liked the illustrations, speed of use, interface colours and the app’s ability to automatically capture images. In addition to their perception that it facilitated a more accurate evaluation of jaundice than current practices, some participants highlighted the advantage of reducing unnecessary blood tests to confirm bilirubin levels. This reduction would minimise the need to prick newborns and would save hospital resources.

While some participants experienced difficulties in using Picterus JP the first time, mainly in initiating image capture, most succeeded after a couple of attempts. During the device’s testing on a dummy, common questions arose regarding its usability in real scenarios. One urban nurse expressed doubts, saying, ‘*Actually, the app is very easy to use … but I think it could be a bit challenging if the baby is moving, making it difficult to capture the images. However, newborns typically don’t move so much’.*

Internet connectivity emerged as a frequent concern regarding the technology’s functioning. Most participants assumed the device’s effectiveness and accuracy, but one mentioned the importance of Picterus JP working properly on all skin tones. The participants shared a concern about cost, believing that the economic factor would be crucial for health authorities considering the inclusion of the device in the already strapped IMSS system. A rural nurse emphasised this concern, stating, *‘[Adoption of the device] could be difficult due to the costs. If IMSS does not have enough money for the catheters we use every day, an app—I don´t know how expensive it could be, but maybe, due to costs, IMSS could not buy it’.*

### B. implementation considerations in the external setting: national health system, IMSS and Oaxaca community

The health administrators considered Picterus JP to be aligned with the mission of the priority national neonatal care programme, as it supports the identification of high-risk newborns and prevents complications related to NNJ, especially in vulnerable areas. However, they acknowledged that implementing a new device within the IMSS involves a lengthy, challenging process. Before it can be used by healthcare providers, a device must obtain approval from the Mexican regulatory body, the Federal Commission for the Protection against Sanitary Risks (COFEPRIS). The acquisition of medical equipment within the IMSS is centrally managed based on the needs and requests of various regions. The administrators emphasised that, in this case, the device’s necessity must be recognised by the staff and requested by local authorities, who then forward the request to the central authorities. Irrespective of staff and local authorities’ needs and requests, however, they believed that a cost-benefit analysis demonstrating the app’s value to the institute is paramount for its approval at the central level.

Regarding parents’ reaction and acceptance of taking photos of their child when using Picterus JP, all the participants agreed on the importance of establishing good communication and building a relationship of trust with them. They also highlighted that increasing awareness through widespread dissemination of Picterus JP in the community would facilitate acceptance. Moreover, some participants expressed the view that using new technology could be interpreted as a sign of progress in healthcare. As one urban nurse put it, *‘If you can explain what it is for and the benefits it can bring to their child, most people would think that we are already improving. I believe it would be well accepted’.*

Internet connectivity was identified as a significant concern, especially in rural areas. A rural nurse summarised this concern, saying, *‘The lack of internet would be a barrier, because everyone would have to use their own data, and there are times when there is no internet available at the hospital. Unless the authorities decide to provide Wi-Fi’.*

### C. implementation considerations in the internal setting: IMSS health facilities in the study

We perceived that the physical facilities at the study sites were adequate for implementing Picterus JP, as they had well-equipped specific areas dedicated to neonatal care. However, insufficient internal information and communication technology (ICT) infrastructure was again emphasised as a barrier to implementation. One health administrator stated, ‘*The only difficulty I find is internet connectivity. We have an institutional network but to a lesser extent than is needed to have a network throughout the hospital, and it is not open; there are restrictions.’*

Regardless of internet connectivity, the compatibility of Picterus JP with routine work and its potential to improve neonatal care were identified as essential factors for the device’s perceived acceptance by HCWs. A family medicine doctor mentioned, ‘*The device is fabulous, timely, effective. … It would not interfere with my daily routine, and it could help me a lot’*. The participants noted that the equipment required to use Picterus JP is minimal, and, in general, they considered the device easy to use.

### D. implementation factors linked to individuals

Regarding implementation factors linked to individuals, all the participants expressed willingness and enthusiasm to learn more about NNJ and to have an additional tool to support its detection. They agreed that Picterus JP is an easy-to-use device that fits well into their work routine. They found it motivating to use new technologies to improve their performance, as stated by an urban nurse: *‘I feel that it is a very useful tool … very accessible and not difficult to use. It would be very useful to detect abnormal cases’.*

According to participants, the level of digital literacy among most HCWs is high enough to support the use of Picterus JP. However, a young participant noted that older HCWs might find it challenging to use the device, stating, *‘In the new generations, it would be very easy, but we have seen that the older generations have resistance to using new technologies’*.

### IV. Healthcare workers’ expectations of picterus JP

Overall, the study’s participants received Picterus JP positively but suggested some modifications and enhancements to better suit their context and maximise its benefits. General practitioners and nurses expressed a common and essential request for the inclusion of decision-support information alongside the bilirubin results. One rural nurse stated, *‘I think it would be very useful to have something to tell us what to do, what the follow-up should be’*, while an urban nurse suggested, *‘Put a figure, like a traffic light, saying “This is normal, this must be monitored and this is for immediate attention”’.*

They also requested learning materials for HCWs that could be shared with parents. Furthermore, an urban nurse suggested more detailed instructions on usage, including age limitations and lighting requirements: *‘Perhaps you can add that it can only be used on babies 1 to 14 days old. … also mention the place where it should be used, I mean, what type of lighting is required to make the measurement. … Perhaps also mention how the [calibration] card should be stored, under what conditions, that there is one for each newborn. If we see that it [the card] is already open or something, no longer use it*’.

Additionally, they mentioned the value of printing the results for referrals or blood test requests. As an urban nurse stated, *‘[It would be an enhancement] to be able to print the result, because, if we refer the baby, maybe they [doctors or laboratory staff] could question us how we got the result.*’ Finally, to address internet connectivity concerns, most participants suggested an offline version of the app as a solution.

## Discussion

This study explored the current state of NNJ detection in rural and urban public health facilities in Oaxaca, the needs and desires of HCWs to improve detection and the stakeholders’ perceptions of Picterus JP, allowing us to gain a broader view of the barriers and facilitators that must be considered when planning the implementation of this innovation in the local health system. The findings reveal deficiencies in the current NNJ detection process and a general desire among the participants for a more accurate method. Ease of use, usefulness and the compatibility of Picterus JP with the work routine were identified as relevant facilitators for implementation, while internet deficiencies and costs were highlighted as the main barriers.

The observations and interviews indicate the relevant role of nurses in providing neonatal care and the challenges they face in detecting NNJ, revealing several deficiencies in this process at the first level of care. Most HCWs, especially nurses, mentioned a lack of updated knowledge and training about NNJ. Similar findings of inadequate knowledge or misconceptions about NNJ among primary HCWs have been reported in other LMICs [34, 35]. Mexican CPGs for the diagnosis and treatment of NNJ were updated in 2019 and are publicly available [13]. However, we observed that frontline workers responsible for NNJ detection often lacked knowledge of them or were not fully aware of their content. A notable example is that all the participants still mentioned unfiltered sun exposure for newborns as a preventive and/or treatment measure for NNJ. This practice is no longer recommended in the national and most international CPGs due to potential side effects, such as exposure to ultraviolet radiation and hyperthermia. This is consistent with previous findings in LMICs, where unfiltered sun exposure and other unsafe practices remain the common advice given by HCWs and among the general population [36].

At the study sites, adherence to Mexico’s CPGs, which recommend measuring bilirubin levels using either TcB or TSB in the presence of visible jaundice, is generally lacking. Instead, the evaluation of newborns heavily relies on HCWs’ subjective assessments of the extent of ‘yellowness’ in the baby’s skin. Similar findings have been reported in a prospective cohort study of 860 Dutch newborns assessed in primary care birth centres [37]. The research revealed that TcB or TSB measurements were not quantified in 44% of newborns considered ‘quite yellow’ and in 20% considered ‘very yellow’. Additionally, the study confirmed that VA was unreliable to estimate TSB levels.

Lack of adherence to CPGs has been identified as a major factor contributing to the persistence of severe NNJ consequences in both LMICs and HICs. For example, a study in Sweden [38] reports that 11 of 13 kernicterus cases identified in the study might have been avoided had the recommendations in the guidelines been followed. A national audit yielded similar findings in the Netherlands [39]. The factors contributing to non-compliance with CPGs have been widely addressed in several systematic reviews [40–42]. The most mentioned are lack of knowledge/awareness/familiarity regarding the guidelines, which is consistent with our findings. Other factors include self-confidence, disagreement with the recommendations, limited work time and the length or complexity of guideline documents. Recommendations to overcome these barriers include dissemination of guideline materials, education and training on their content, access to relevant guidelines at the point of care and regulatory and financial incentives [41, 42].

In addition to non-compliance with CPGs, we found that NNJ detection was usually performed in a non-systematic way at the study sites and that HCWs lacked a tool to support their screening, resulting in suboptimal confidence among most participants about their own NNJ evaluation. At the study sites, no baseline data were found on the prevalence of NNJ, its consequences or the number of newborns referred for further evaluation based on VA. However, all newborns were scheduled for neonatal metabolic screening and immunisation three to seven days after birth, usually coinciding with the presence of NNJ. According to the interviewed health administrators, more than 70% of mothers attend these appointments, representing a significant opportunity to identify newborns at risk of developing severe hyperbilirubinemia. Similar findings were reported in a retrospective study in which 60% of newborns admitted to a Mexican hospital with diagnosis of NNJ were identified by VA when they visited the preventive medicine unit for neonatal metabolic screening [43]. Therefore, implementing educational programmes with simple, easy-to-understand learning materials to raise awareness of the NNJ guidelines in these units could positively impact its detection. Equally relevant is training HCWs to carry out VA in a systematic way (supported, for example, with printed charts) and providing more reliable detection tools, such as Picterus JP. There is strong evidence from LMICs that the combination of education and training for HCWs and family members along with access to proper detection and treatment equipment improves the outcomes of newborns affected by NNJ [44].

Picterus JP was perceived by all participants as a device that is easy to use, useful and compatible with work routines. Several authors have considered such positive perceptions and attitudes among stakeholders as determining factors for the acceptance and adoption of mHealth innovations by HCWs [45–49]. Contrarily, researchers have found that HCWs feel frustrated and unwilling to use mobile devices when the apps delay the workflow or are not easy to use [46]. It is important to remark that Picterus JP was tested on a dummy, which may differ from real-life use. Prior validation studies, including the pilot study in Mexico, have shown a high correlation with TSB (Pearson’s correlation coefficient between 0.84 and 0.87) and high sensitivity (between 85 and 94%) in detecting severe NNJ, defined as TSB ≥ 250 µmol/L, in newborns with light to moderate brown skin tone [17, 19]. However, introducing a new device into clinical practice requires more than demonstrating technical accuracy. Further studies in the setting using the device on newborns are required to determine its usability more precisely in a real-world context.

Extensive evidence shows that multi-stakeholder engagement in the development and adaptation of mHealth systems is essential for successful scale-up and implementation [50–52]. Picterus JP’s development followed an iterative process in which end users tested the device on a dummy and on newborns and then offered feedback to optimise the system [17]. The device has also been tested on newborns by 61 Dutch maternity nurses in their routine home visits to monitor NNJ in both urban and rural regions (unpublished results). Most of them felt that Picterus JP supported their work, and 56% found the device easy to use. A common request among users was to add supporting information for deciding when to refer a newborn for a blood sample or further evaluation. Although these results are from HICs, they are similar to our findings and reflect frontline workers’ great need for decision support and knowledge of NNJ in general. Since most of our participants were not familiar with CPGs, the bilirubin levels displayed on the device held no meaningful significance for them. Therefore, having decision-making support is essential for Picterus JP to provide tangible value in practice and, consequently, enhance care for newborns. This aligns with the opinion of World Health Organisation experts that the use of decision-support tools on mobile devices can improve care delivery and increase efficiency and compliance with guidelines [53].

This study’s main purpose was to use contextual observations and stakeholder perspectives to identify the barriers and facilitators to implementing Picterus JP as a supporting tool to detect NNJ in low-resource Mexican healthcare facilities. Several challenges to the successful implementation of the device were identified. As previously described in LMICs [45, 46, 54, 55], factors related to information and communication technology (ICT) infrastructure, policy/regulatory aspects and costs were highlighted as the most relevant barriers to address when planning implementation strategies for the device.

Inaugurated by the government in 2013, Mexico’s National Digital Strategy (Estrategia Digital Nacional) includes the aim of taking advantage of ICT to improve health service quality, coverage and effective access as well as to more efficiently use health resources and infrastructure [56]. Recent years have brought great advances in health technology in the country, predominantly in large cities and at the private level [57], but poor internet coverage and deficiencies in mobile network provision are common factors that hinder access to new technologies in several rural areas [58]. This technological gap has been attributed to obsolete infrastructure, lack of maintenance and geographical factors affecting the country’s most vulnerable populations [59]. Picterus JP was developed to be a globally relevant, affordable and accessible device to support NNJ detection, particularly in settings lacking reliable screening tools. The current version of the device depends on internet connectivity, which hinders the accomplishment of that goal. Therefore, to succeed, it is essential to develop a version that works with reduced or no internet connection.

To introduce a new medical device into the Mexican healthcare system, its safety and efficacy must first be approved by the regulatory body COFEPRIS. Furthermore, it is necessary to demonstrate the cost-effectiveness of the device for public health institutions, which would require funds for procurement. This process is typically lengthy and cumbersome, often acting as a barrier to implementing innovations in the health system. Picterus JP has strong scientific support for its safety and efficacy but overcoming the financial issue is more difficult. Mexico faces significant and varied challenges in terms of healthcare services. The population lacking access to health services grew from 16.2% in 2018 to 28.2% in 2020. In addition, the health sector budget has been consistently below 3% of the gross domestic product, whereas the international suggestion is to allocate more than 6% [60]. Our participants perceived that sometimes they lacked even basic resources for their daily work. Exploratory and feasibility studies are essential for obtaining an accurate understanding of the context and developing a comprehensive and multilevel implementation strategy. Coordination and collaboration among several partners and key stakeholders in the public and private sectors of the economy, including multinational and non-governmental organisations, will be crucial to achieving successful outcomes in the implementation of this mHealth device in the Mexican health system.

### Strengths and limitations

The use of qualitative methods in this study provided an in-depth, contextual understanding from diverse perspectives of the current deficiencies in general knowledge about NNJ and its detection as well as what the participants require to bridge this gap. We also obtained sufficient and valuable information to optimise Picterus JP so that it can be tailored to become a potentially useful and reliable supporting tool for HCWs dedicated to neonatal care. Additionally, using the CFIR to guide our analysis enabled the systematic identification and evaluation of relevant barriers and facilitators that merit consideration when planning implementation strategies. There are no precedents of similar studies that address NNJ and the use of mHealth to support its detection at the local or national levels in Mexico, so our findings may provide important information to increase awareness of NNJ care among HCWs, health administrators and policymakers. Furthermore, the study can be useful in the development and implementation of other types of mHealth devices in the same or similar contexts.

The study has some limitations. First, only two IMSS study sites in one specific region in Oaxaca were included. Therefore, our findings cannot be generalised to HCWs or healthcare facilities located in other regions or managed by a different healthcare provider. Second, the interviews were conducted in consultation offices at the workplace, and, although we tried to maintain privacy, noises from the outside were audible, and we were sometimes interrupted by other HCWs. This may have made our participants uncomfortable and/or distracted, potentially limiting their responses. Third, it would have been ideal to interview more health administrators or authorities at higher levels of the organisation to obtain a more accurate picture of the feasibility of implementing the device in the IMSS; however, due to time constraints on their part, we could include only two of them. The limited representation of certain HCWs reflects real-world availability and accessibility at the study sites. Finally, Picterus JP was tested on a dummy, so further studies are needed in real-world settings.

### Future studies

Based on the findings of this study, Picterus JP needs to be optimised and field tested among HCWs to assess the device’s usability and feasibility in a real scenario. The impact of the device on newborns’ health outcomes compared to the current detection method needs to be quantitatively assessed, and a cost-benefit analysis of implementing the device in the health system is needed.

There is also a potential for Picterus JP to be used by parents to screen their infants at home. A quantitative pilot usability test using a questionnaire with 96 parents was previously conducted at our study sites [61]. The results show that the overall experience of using the device was favourable; however, the potential of the device to assess the newborn was better rated than its usability, specifically, its ease of use. Another relevant finding was that 77% of the participants lacked or had only minimal knowledge of NNJ. Further studies are needed to qualitatively assess parents’ knowledge of NNJ and their experiences and potential benefits of using the device at home.

## Conclusions

Picterus JP and mHealth tools in general have the potential to facilitate and enhance the work of frontline HCWs in LMICs; however, contextual factors must be considered in their design, development and implementation to maximise the potential benefits they are intended to provide. Technical infrastructure and financial barriers are the main challenges to introducing Picterus JP in the Mexican healthcare system and must be addressed for successful implementation outcomes. In addition, there is scope to strengthen the current process of NNJ detection through specific educational and training programs targeting HCWs who provide neonatal care to increase awareness and knowledge of the condition and improve the VA of newborns.

### Electronic supplementary material

Below is the link to the electronic supplementary material.

Additional file 1: Guideline for semi-structured interviews and focus group for health care workers and health administrators after testing the current version of Picterus JP in a dummy

Additional file 2: Identified codes and their corresponding CFIR domain and construct

## Data Availability

The datasets generated and/or analysed during the current study are not publicly available to maintain anonymity and protect participants’ confidentiality but are available from the corresponding author on reasonable request.

## Abbreviations

NNJ: Neonatal jaundice
VA: Visual assessment
Picterus: JP Picterus Jaundice Pro
TcB: Transcutaneous bilirubinometer
TSB: Total serum bilirubin
LMICs: Low-and middle-income countries
HICs: High-income countries
CPGs: Clinical practical guidelines
NICU: Neonatal intensive care unit
HCWs: Health care workers
IMSS: Mexican Institute of Social Security
CFIR: Consolidated framework for implementation research
ER: Emergency room
COFEPRIS: Federal commission for the protection against sanitary risks
ICT: Information and communication technologies
