# Supplementary material for: Neonatal jaundice detection in low-resource Mexican settings: possibilities and barriers for innovation with mobile health
Source: BMC Health Serv Res. 2024 May 28;24:671. doi: 10.1186/s12913-024-11141-6 (PMC11134921; doi:10.1186/s12913-024-11141-6)
Supplement: Supplementary file 1 — Additional file 1: Guideline for semi-structured interviews and focus group for health care workers and health administrators after testing the current version of Picterus JP in a dummy [file 12913_2024_11141_MOESM1_ESM.docx]

Objective: Identify user’s experience of the current version of *Picterus*. Identify attitudes toward acceptance and perceived barriers to introducing *Picterus* into the local health care system**.**

**General data**

1. Can you please tell me your education background/profession, age, years of experience and time you have been working in this ward?

**General perceptions**

1. In general, how do you feel about using the app?
   1. How did you experience using the app?
2. How user-friendly do you find the app?
   1. In general, how easy did you find to use it?
   2. What do you think about the initial instructions?
   3. Which parts of the app did you experience as easy?
   4. Which parts of the app did you experience as difficult?
   5. Could you easily find what you need?
   6. Were there moments that you got stuck?
   7. Could you easily read all the texts?

**Time-efficiency**

1. What to do think about the time you needed to complete a screening with the app?
   1. How can this time interfere with your current tasks?
2. To what extent do you think using the app can help you to improve NNJ detection?

**Lay out**

1. What do you think about the design of the app?
   1. Which parts of the app did you like the most?
   2. Which parts of the app did you like the least?
   3. Do you suggest any adaptations/changes/additions of the app?
   4. What do you think about including NNJ training and information in the app?

**Perceptions about adoption of the app**

1. How easy or difficult do you think this app would be accepted to use by health care workers?
2. How easy or difficult do you think the app could be introduced and adopted in your workplace?

**Perceptions about parents use**

1. What do you think about the use of the app at home by parents?
   1. Do you think it would be easy to use for them?
   2. Do you have any suggestions about how the app can be used by parents?
