# Supplementary material for: Neonatal jaundice detection in low-resource Mexican settings: possibilities and barriers for innovation with mobile health
Source: BMC Health Serv Res. 2024 May 28;24:671. doi: 10.1186/s12913-024-11141-6 (PMC11134921; doi:10.1186/s12913-024-11141-6)
Supplement: Supplementary file 2 — Additional file 2: Identified codes and their corresponding CFIR domain and construct [file 12913_2024_11141_MOESM2_ESM.docx]

| **I. INNOVATION DOMAIN**  **Innovation: Picterus JP app to detect neonatal jaundice** | |
| --- | --- |
| **Construct Name and definition (degree to which:)** | **IDENTIFIED CODES or CURRENT FACTS** |
| - Innovation Evidence-Base   The innovation has robust evidence supporting its effectiveness. | Picterus JP has been tested in clinical trials |
| - Innovation Relative Advantage   The innovation is better than other available innovations or current practice. | Picterus JP has shown higher accuracy than current practice (visual assessment) |
| - Innovation Adaptability   The innovation can be modified, tailored, or refined to fit local context or needs. | Suggested additions and improvements to Picterus JP |
| - Innovation Trialability   The innovation can be tested or piloted on a small scale and undone. | Picterus JP can and will be piloted |
| - Innovation Complexity   The innovation is complicated, which may be reflected by its scope and/or the nature and number of connections and steps. | Perceived ease of use |
| - Innovation Design    The innovation is well designed and packaged, including how it is assembled, bundled, and presented. | Satisfaction with Picterus JP design and instructions |
| - Innovation Cost   The innovation purchase and operating costs are affordable. | Perceived adoption of Picterus JP by IMSS |
| **II. OUTER SETTING DOMAIN**  **Outer Setting: Health facilities belonging to the public health provider Instituto Mexicano del Seguro Social** | |
| **Construct Name** | **IDENTIFIED CODES or CURRENT FACTS** |
| - Critical Incidents   Large-scale and/or unanticipated events disrupt implementation and/or delivery of the innovation. | Picterus JP should be first approved by Mexican Regulations, and then approved by IMSS administrators  Perceived adoption of Picterus JP by IMSS |
| - Local Attitudes   Sociocultural values (e.g., shared responsibility in helping recipients) and beliefs (e.g., convictions about the worthiness of recipients) encourage the Outer Setting to support implementation and/or delivery of the innovation | Perceived acceptance of Picterus JP by parents |
| - Local Conditions   Economic, environmental, political, and/or technological conditions enable the Outer Setting to support implementation and/or delivery of the innovation. | Perceived adoption of Picterus by IMSS  Recent health policies promote the use of health technologies |
| - External Pressure: Societal Pressure   Mass media campaigns, advocacy groups, or social movements or protests drive implementation and/or delivery of the innovation. | Perceived acceptance of Picterus JP by parents |
| **III. INNER SETTING DOMAIN**  **Inner Setting: Family Medicine Unit 65, Rural Hospital Tlacolula** | |
| **Construct Name** | **IDENTIFIED CODES or CURRENT FACTS** |
| - Structural Characteristics - Physical Infrastructure   Layout and configuration of space and other tangible material features support functional performance of the Inner Setting.   - Information Technology Infrastructure   Technological systems for tele-communication, electronic documentation, and data storage, management, reporting, and analysis support functional performance of the Inner Setting   - Work Infrastructure .   Relational Connections | OBS: clinic and hospital facilities are suitable places to implement the app  Perceived adoption at workplace  OBS: formal and informal relationships, networks and teams are suitable for implement the app |
| - Culture   There are shared values, beliefs, and norms across the Inner Setting. | Perceived adoption of Picterus JP by HCW |
| - Compatibility   The innovation fits with workflows, systems, and processes. | Perceived speed of use, perceived usefulness  workload, NNJ current detection process |
| - Relative Priority   Implementing and delivering the innovation is important compared to other initiatives. | Need for NNJ detection support and improvement.  Perceived usefulness |
| - Mission Alignment   Implementing and delivering the innovation is in line with the overarching commitment, purpose, or goals in the Inner Setting. | Workload, newborn care, NNJ current detection process |
| - Available Resources - Materials & Equipment   Supplies are available to implement and deliver the innovation. | Perceived adoption of Picterus JP by IMSS  Perceived adoption of Picterus JP by HCW |
| **IV. INDIVIDUALS DOMAIN**  **Individuals:** **HCW delivering neonatal care, health administrators**   - **Characteristics subdomain** | |
| **Construct Name** | **IDENTIFIED CODES or CURRENT FACTS** |
| - Need   The individual(s) has deficits related to survival, well-being, or personal fulfillment, which will be addressed by implementation and/or delivery of the innovation. | Perceived burden of NNJ  Perception about NNJ detection  Need for knowledge and training  Need for NNJ detection improvement |
| - Capability   The individual(s) has interpersonal competence, knowledge, and skills to fulfill Role.   - Opportunity   The individual(s) has availability, scope, and power to fulfill Role.   - Motivation   The individual(s) is committed to fulfilling Role. | Self-efficacy  Perceived usefulness  Perceived adoption by HCW  Observed motivation to use the app |
